# Supplementary material for: Time-varying optimization of COVID-19 vaccine prioritization in the context of limited vaccination capacity
Source: Nat Commun. 2021 Aug 3;12:4673. doi: 10.1038/s41467-021-24872-5 (PMC8333090; doi:10.1038/s41467-021-24872-5)
Supplement: Supplementary file 1 — Supplementary Information [file 41467_2021_24872_MOESM1_ESM.pdf]

Time-varying optimization of COVID-19 vaccine prioritization in the context of limited vaccination capacity

Supporting Information

Shasha Han[1,2#], Jun Cai[3#], Juan Yang[3,4], Juanjuan Zhang[3], Qianhui Wu[3], Wen Zheng[3], Huilin Shi[3], Marco Ajelli[5,6†], Xiao-Hua Zhou[1,7,8†*], Hongjie Yu[3,4,9†*]

1. Beijing International Center for Mathematical Research, Peking University, Beijing, China
2. Harvard Medical School, Harvard University, Boston, MA, USA.
3. School of Public Health, Fudan University, Key Laboratory of Public Health Safety, Ministry of Education, Shanghai, China
4. Shanghai Institute of Infectious Disease and Biosecurity, Fudan University, Shanghai, China
5. Department of Epidemiology and Biostatistics, Indiana University School of Public Health, Bloomington, IN, USA
6. Laboratory for the Modelling of Biological and Socio-technical Systems, Northeastern University, Boston, MA, USA
7. Department of Biostatistics, School of Public Health, Peking University, Beijing, China
8. National Engineering Laboratory of Big Data Analysis and Applied Technology, Peking University, Beijing, China
9. Department of Infectious Diseases, Huashan Hospital, Fudan University, Shanghai, China

[#]These authors contributed equally.

[†]These authors are joint supervised this work.

*To whom correspondence should be addressed to:

X.-H. Z. (azhou@math.pku.edu.cn) and H.Y. (yhj@fudan.edu.cn).

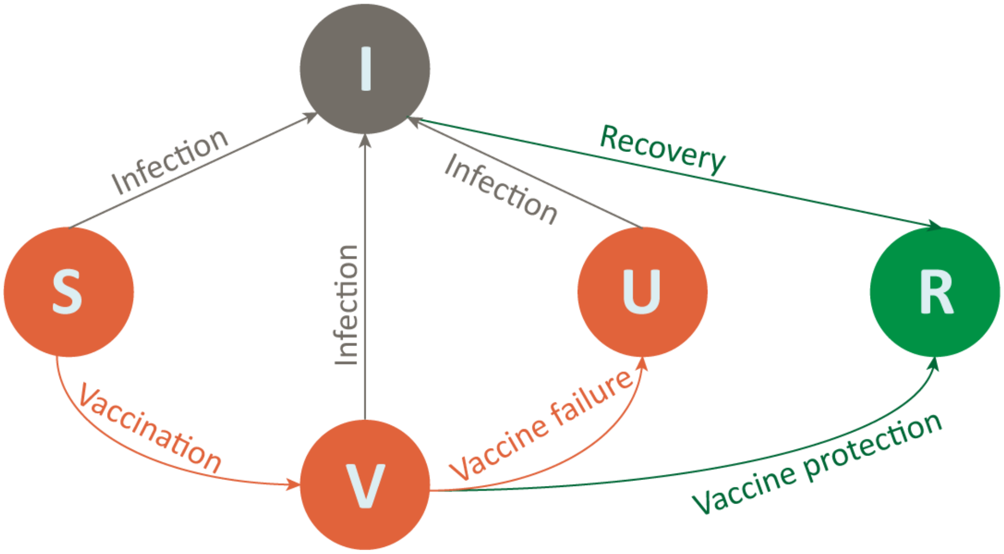

Supplementary Fig. 1: Schematic representation of the transmission model. By omitting the dependency on age and time, model compartments are defined as follows: S represents unvaccinated susceptible individuals; V represents individuals who have received the vaccine but have yet to develop protection; U represents individuals who has received the vaccine(s) but fail to get protection; I represents infectious individuals; R represents individuals who are immune to the infection either due to recovery after natural infection or successful vaccination. The diagram applies to all-or-nothing model (Equation (1), used in the main analysis).

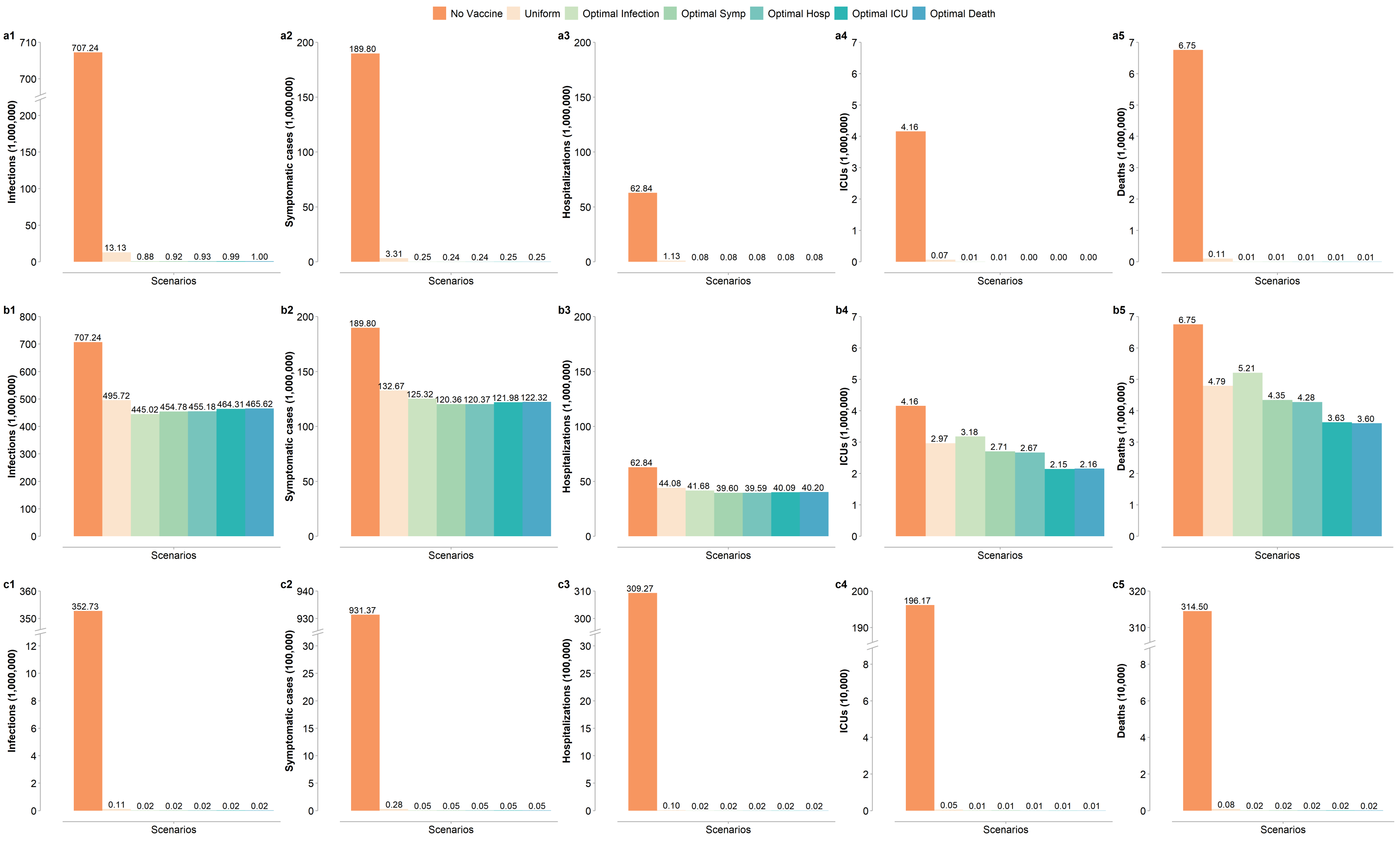

Supplementary Fig. 2: Risk incidences under different capacities and reproduction numbers. a1, Number of infections under 3.5 million (rollout speed 0.24%) and R = 1.5. **b1,** As **a1,** but for 1.0 million first doses per day (rollout speed 0.07%). **c1**, As **a1,** but for R =1.25. **a2**-**a5**, **b2**-**b5** and **c2**-**c5**, As panel **a1**, **b1** and **c1** respectively, but for the number of symptomatic cases, hospitalizations, ICUs, and deaths. The uniform strategy has comparable performances to optimal prioritization strategy on infections. Keys apply to all panels.

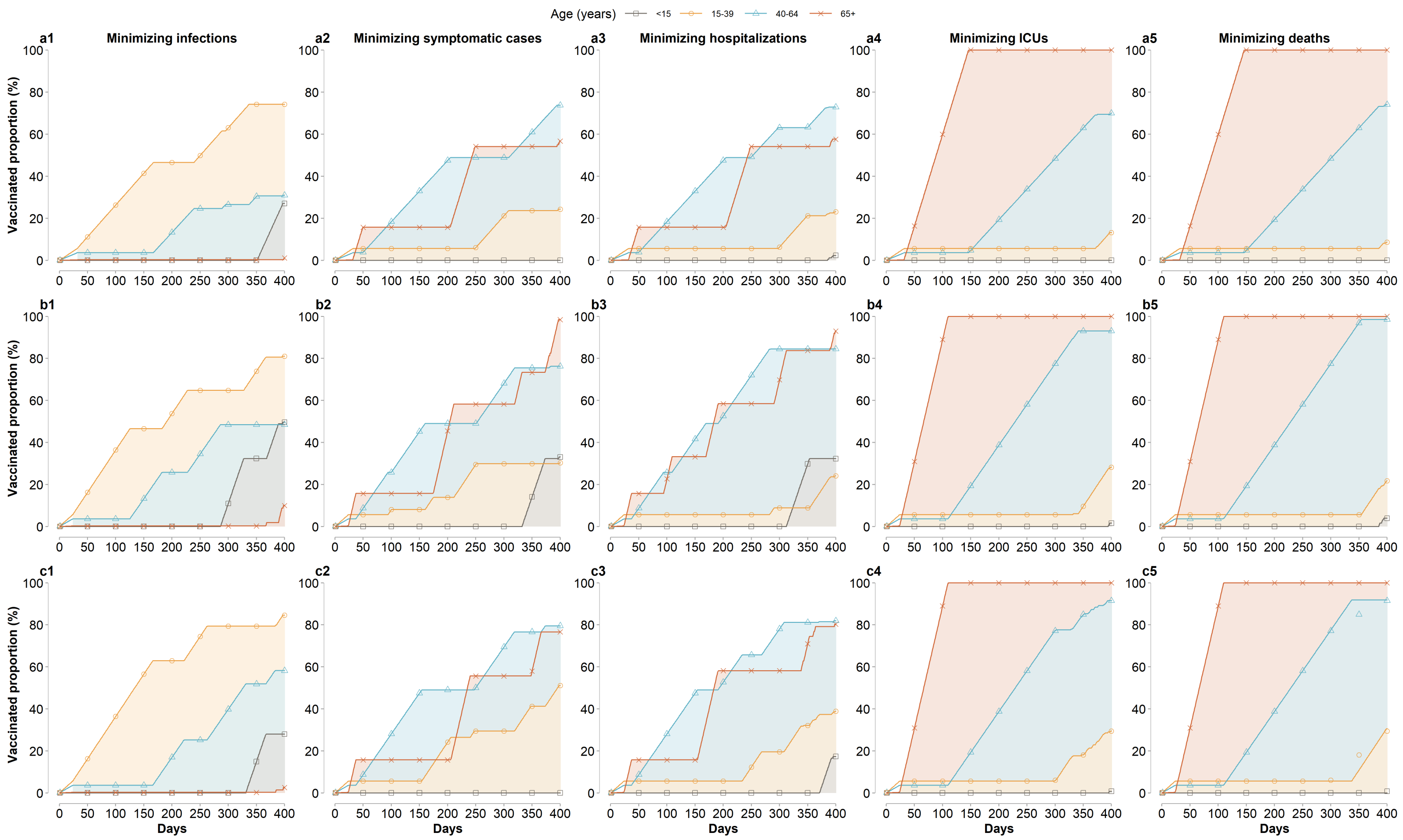

Supplementary Fig. 3: Vaccinated proportions for different prioritizations under three scenarios. a1, Prioritization strategy for minimizing infections, under 80% vaccine efficacy for people aged 15-59 and 80%×0.75 for the rest, and rollout speed 0.10% (1.5 million courses). **b1**, Strategy for minimizing infections with age-specific vaccine efficacy with 80% replaced by 90%, and rollout speed 0.14% (2.0 million courses). **c1**, Strategy for the scenario where vaccines have 60% efficacy preventing infections and 80% preventing symptomatic disease (50% preventing symptomatic disease given infections). **a2-a5**, **b2-b5** and **c2-c5**, As panel **a1**, **b1** and **c1** respectively, but for minimizing symptomatic cases, hospitalizations, ICUs, and deaths. Shaded area refers to vaccine administration to the general populations. Lines refers to the vaccinated proportions including essential workers. Keys and labels apply to all panels.

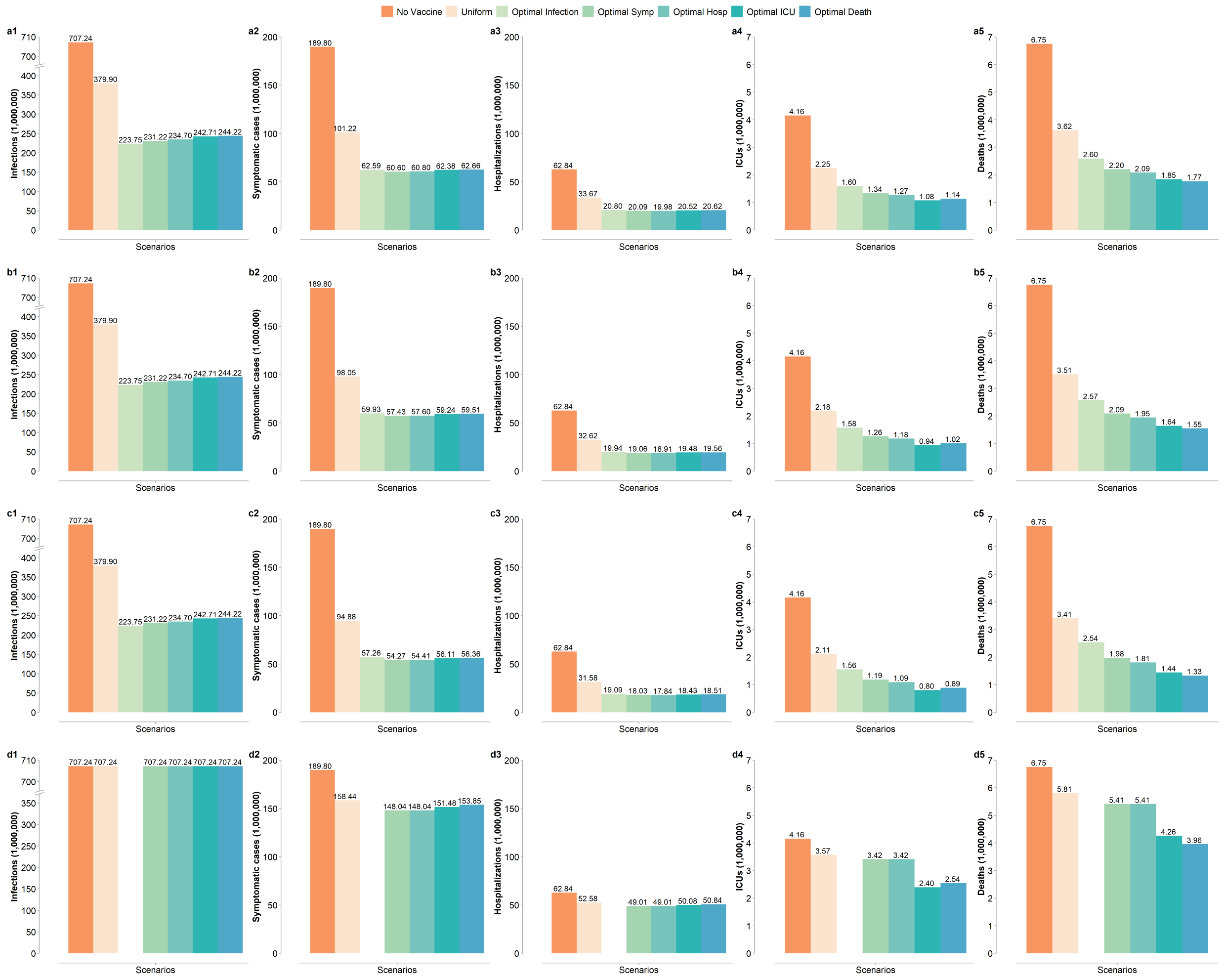

Supplementary Fig. 4: Risk incidences under different scenarios on vaccine efficacy against the infection and disease. a1, Number of infections for the scenario considering 0% additional protection in preventing the disease given the infection. Vaccine efficacy in preventing the infection is fixed at 60% for individuals aged 15-59 years and 60%×0.75 for the other age groups. **b1**, As **a1**, but considering 25% additional protection in preventing the disease given the infection. **c1**, As **a1**, but considering 50% additional protection in preventing the disease given the infection. **d1**, Number of infections for the scenario considering 0% efficacy in preventing the infection and 80% efficacy in preventing the disease in individuals aged 15 -59 years and 80%×0.75 for the other age groups. **a2-a5**, **b2-b5** and **c2-c5**, As panel **a1**, **b1** and **c1** respectively, but for the number of symptomatic cases, hospitalizations, ICUs, and deaths. Keys apply to all panels

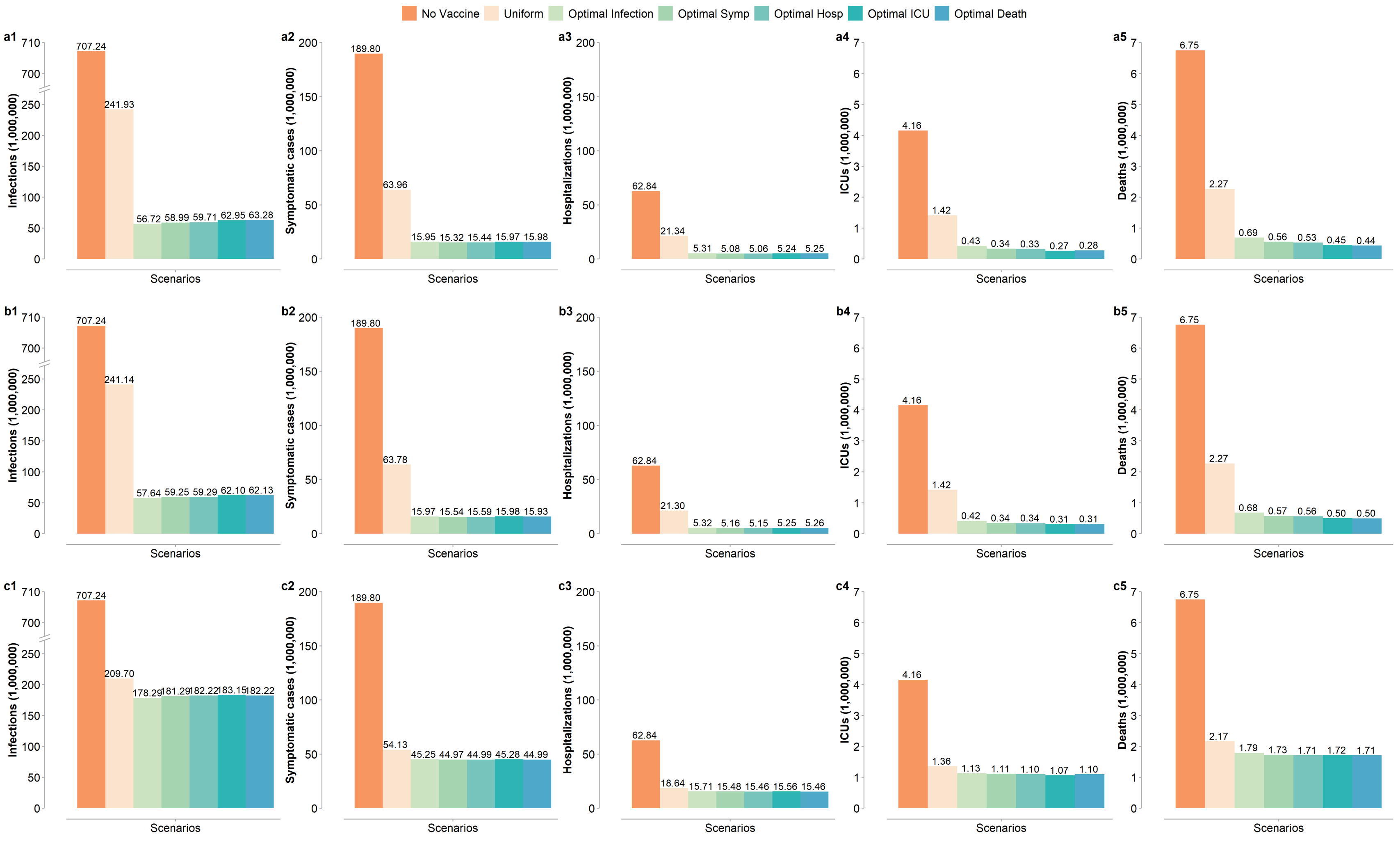

Supplementary Fig. 5: Risk incidences under vaccine hesitancy. a1, Number of infections for "Chinese survey", i.e., on average 83% of the population is willing to accept the vaccine as estimated in a survey conducted on the Chinese population (age-specific estimates reported in Supplementary Table 2). **b1**, As **a1**, but for "Global survey", i.e., on average 61% of the population is willing to accept the vaccine as estimated in a survey at the global level [1]. **c1**, Number of infections when vaccinations excluding people under 20 and adults over 60. The uniform strategy improves remarkably when vaccinations excluding the kids and the old. **a2**-**a5**, **b2**-**b5** and **c2**-**c5**, As panel **a1**, **b1** and **c1** respectively, but for the number of symptomatic cases, hospitalizations, ICUs, and deaths. Keys apply to all panels.

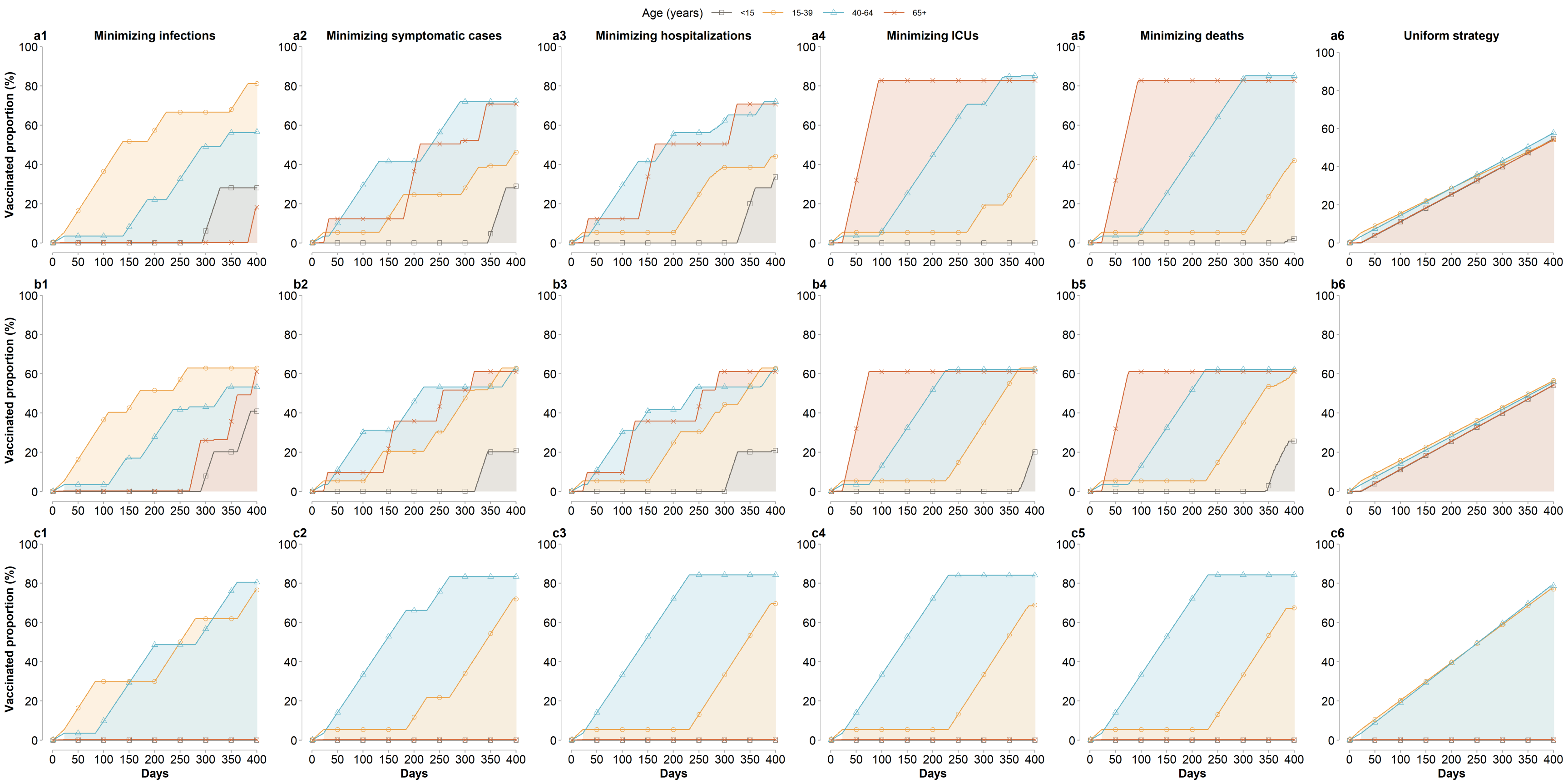

Supplementary Fig. 6: Prioritization strategies under vaccine hesitancy. a1, Optimal prioritization strategy under minimizing infections for "Chinese survey", i.e., on average 83% of the population is willing to accept the vaccine as estimated in a survey conducted on the Chinese population (age-specific estimates reported in Supplementary Table 2). **b1**, As **a1**, but for "Global survey", i.e., on average 61% of the population is willing to accept the vaccine as estimated in a survey at the global level [1]. **c1**, Optimal prioritization for minimizing infections when vaccinations excluding people under 20 and adults over 60. The priority orders are similar to that in the baseline. **a2-a5**, **b2-b5** and **c2-c5**, As panel **a1**, **b1** and **c1** respectively, but for minimizing symptomatic cases, hospitalizations, ICUs, and deaths. The priority orders are similar to that in the baseline. Keys apply to all panels. Shaded area refers to vaccine administration to the general populations. Lines refers to the vaccinated proportions including essential workers.

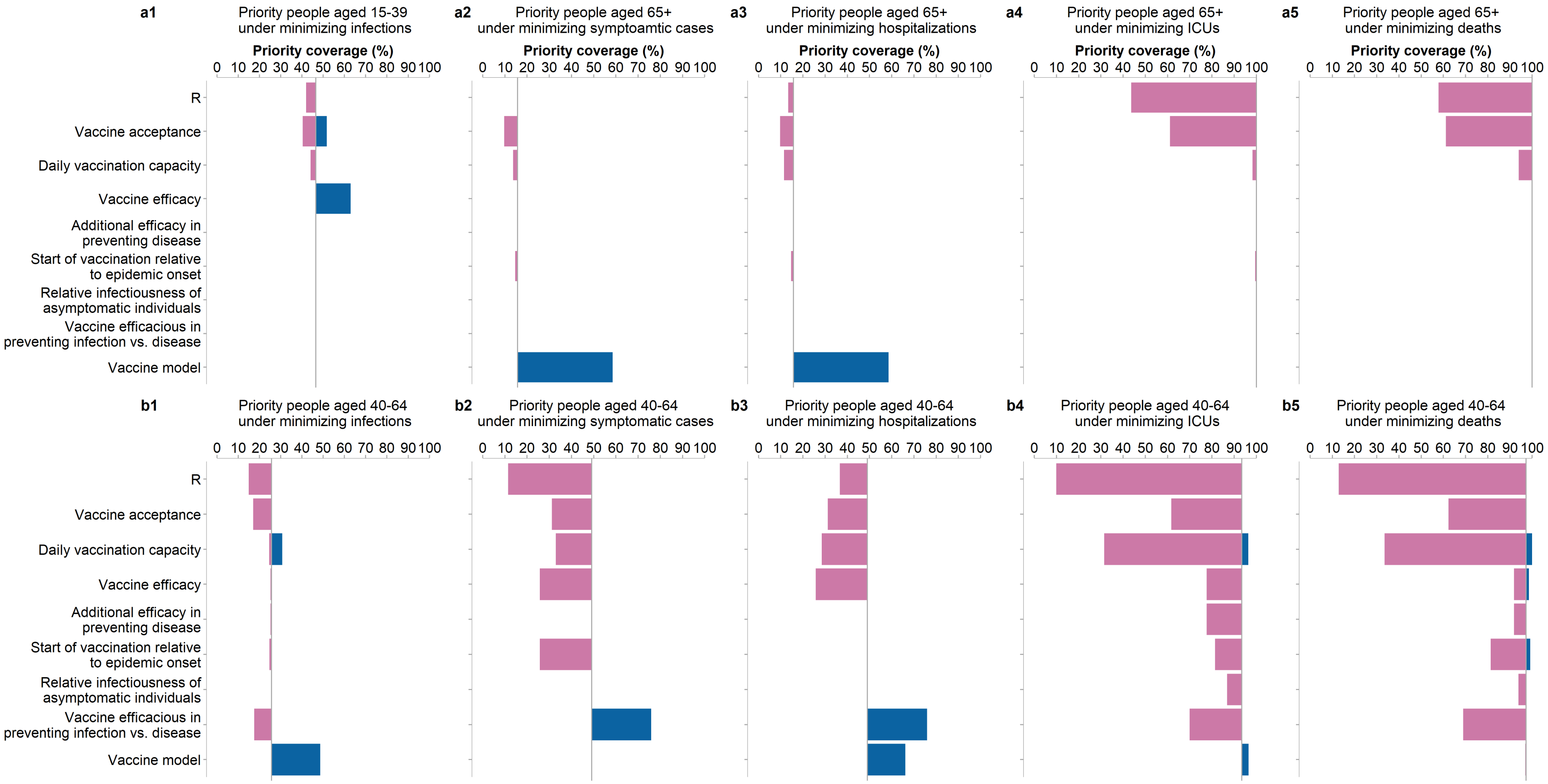

Supplementary Fig. 7: Variations of priority coverages for the first two priority groups. a1-a5, for the first priority group and **b1-b5**, for the second priority groups. Start of vaccination relative to epidemic onset does not include the scenarios where the campaign starts more than 60 days earlier than the epidemic onset. Overall, coverages for the first two priority groups under minimizing infections have small variations across varying scenarios.

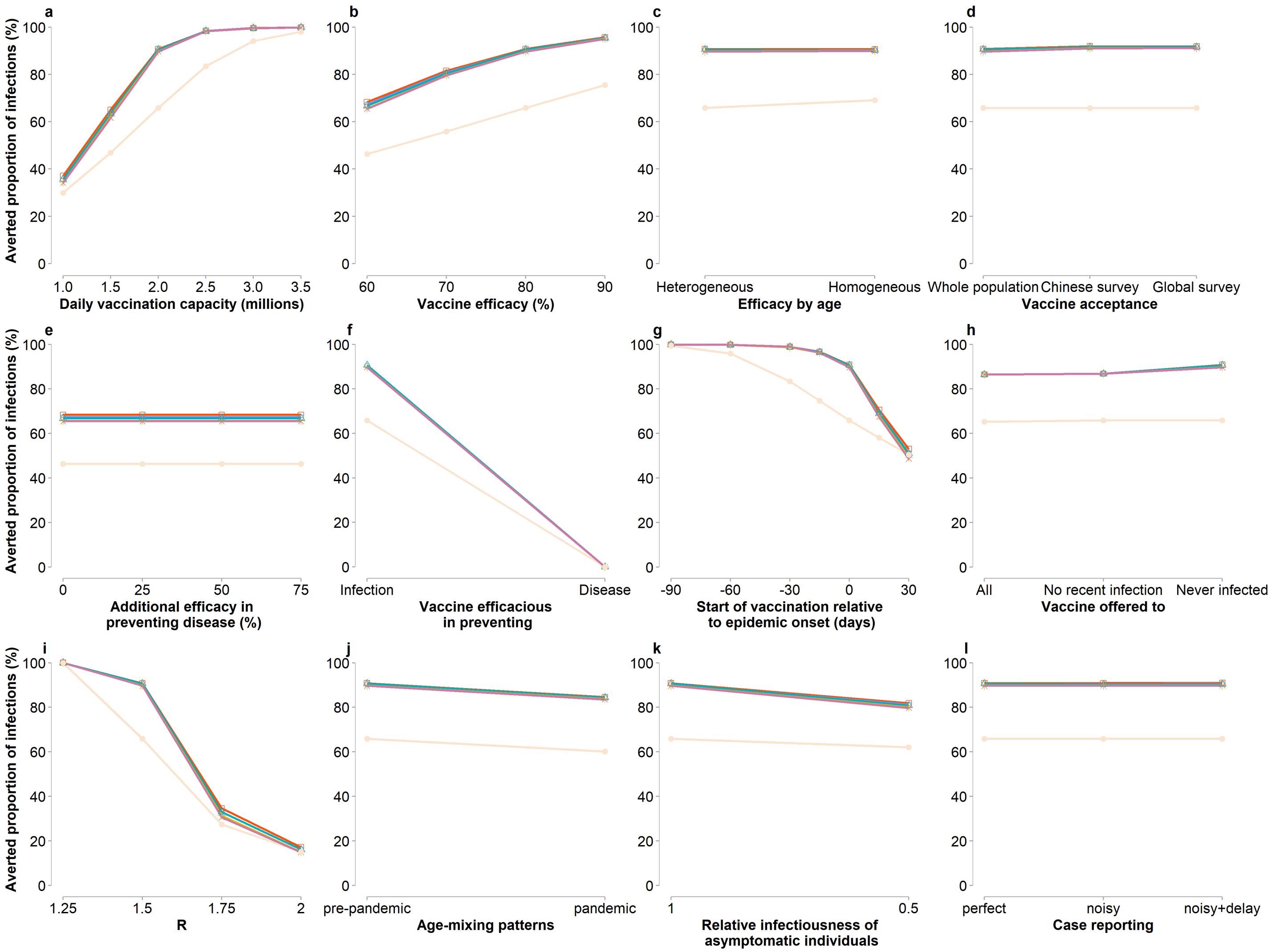

Supplementary Fig. 8: Averted infections in comparison to the scenario with no vaccines. As Fig. 3, but for infections. Patterns are similar to that are observed in Fig. 3. Keys apply to all panels. Labels apply to panels in the same row (column).

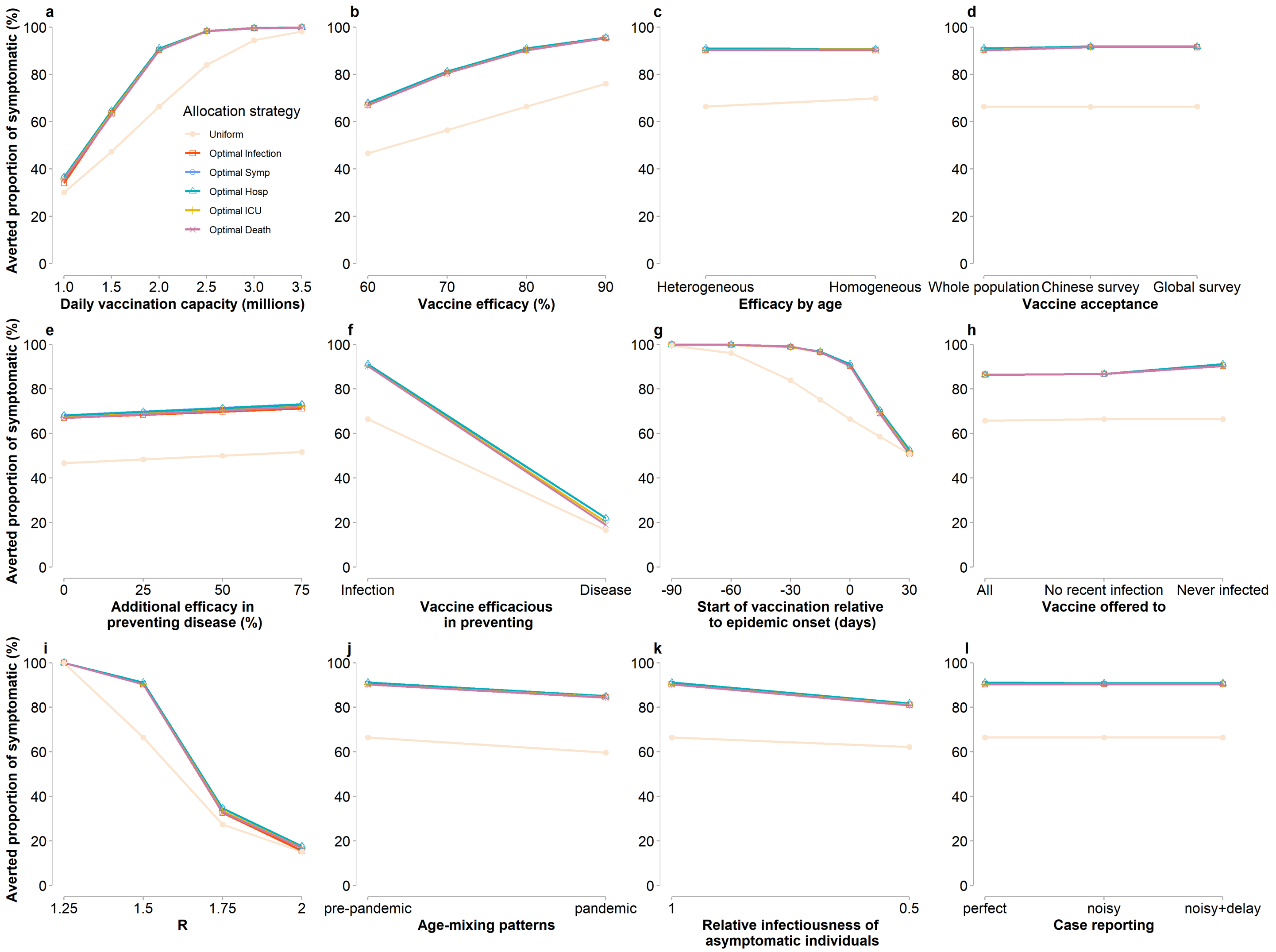

Supplementary Fig. 9: Averted symptomatic cases in comparison to the scenario with no vaccines. As Fig. 3, but for symptomatic cases. Patterns are similar to that are observed in Fig. 3. Keys apply to all panels. Labels apply to panels in the same row (column).

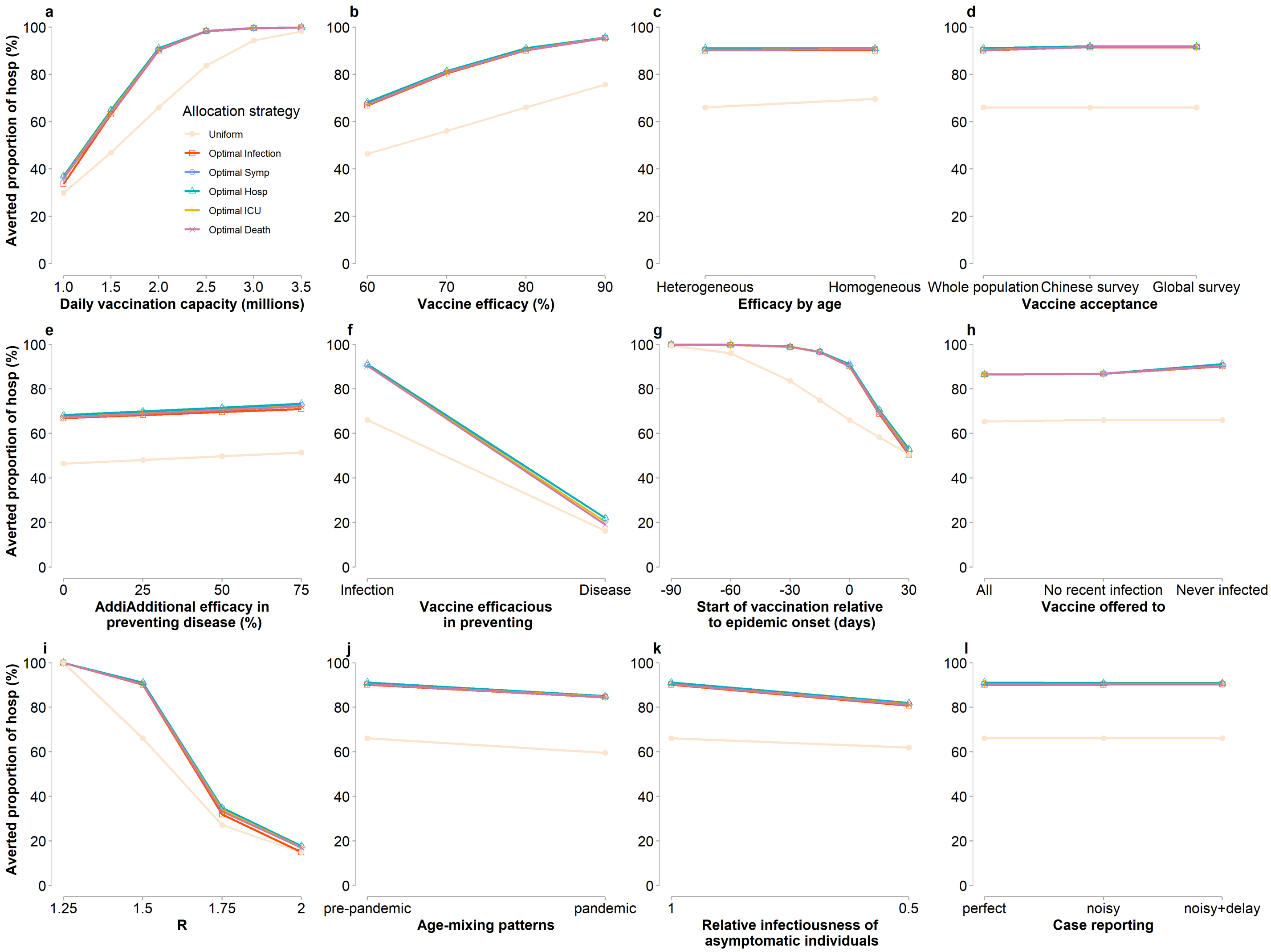

Supplementary Fig. 10: Averted hospitalizations in comparison to the scenario with no vaccines. As Fig. 3, but for hospitalizations. Patterns are similar to that are observed in Fig. 3. Keys apply to all panels. Labels apply to panels in the same row (column).

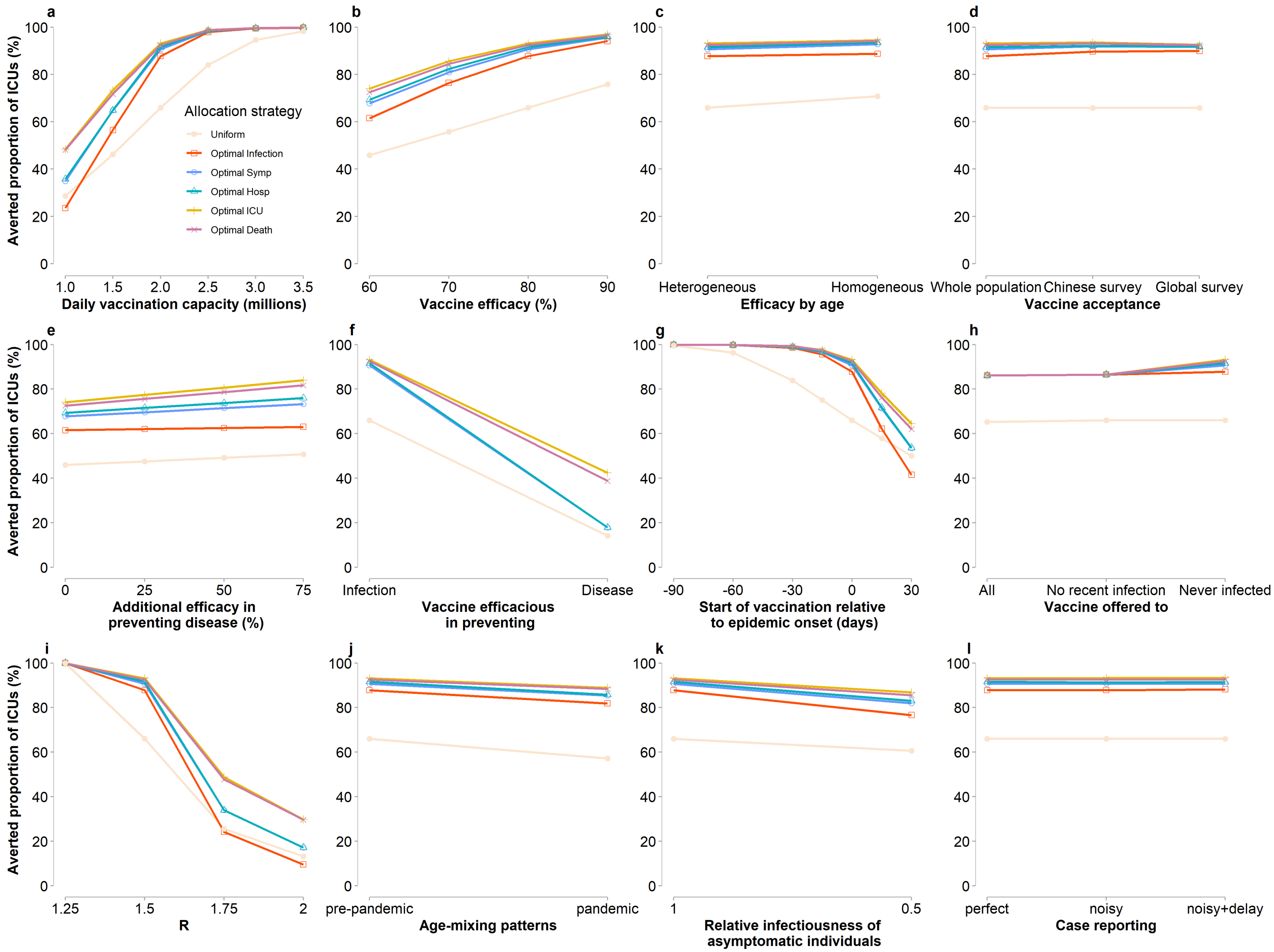

Supplementary Fig. 11: Averted ICUs in comparison to the scenario with no vaccines. As Fig. 3, but for ICUs. Patterns are similar to that are observed in Fig. 3. Keys apply to all panels. Labels apply to panels in the same row (column).

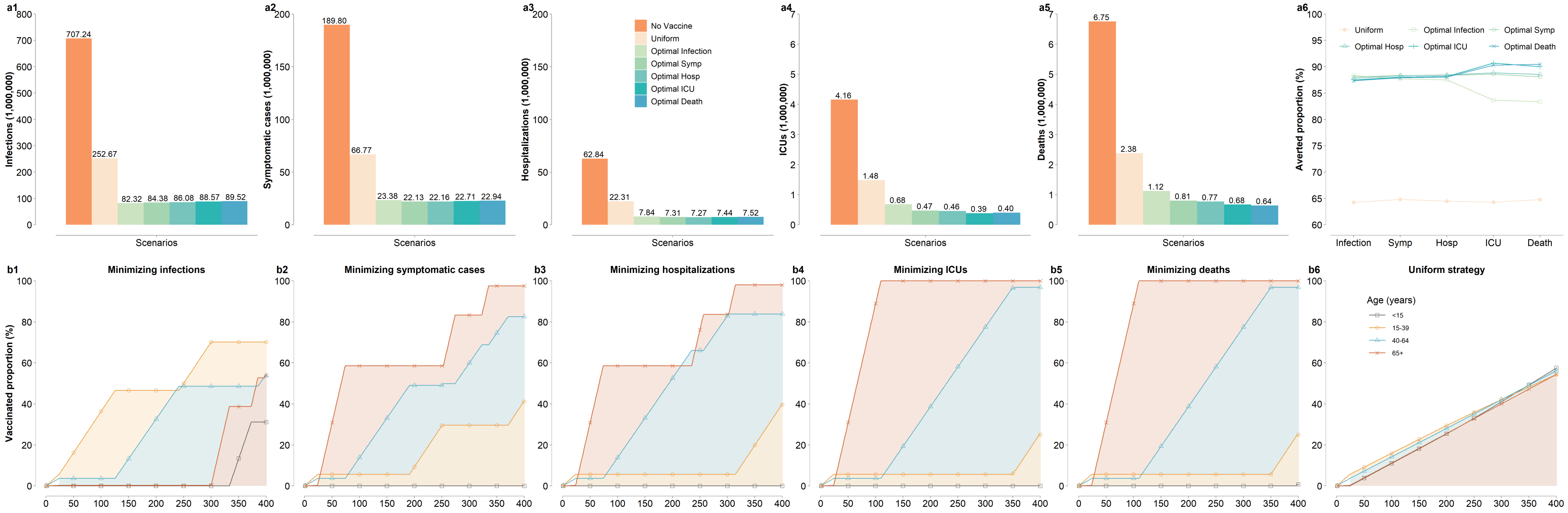

Supplementary Fig. 12: Risk incidences and prioritization strategies assuming a "leaky" vaccine (rollout speed 0.14% and R = 1.5). The "leaky vaccine" model uses 80% vaccine efficacy for people aged 15-59 and 80%×0.75 for the rest, as in Figs. 1 and 2. **a1-a6**, risk incidences and averted proportions under the leaky vaccine model. **b1**, the optimal prioritization strategy for minimizing infections under the leaky vaccine model. **b2-b6**, As panel **b1**, but for minimizing symptomatic cases, hospitalizations, ICUs and deaths, and the uniform strategy. The optimal prioritization strategies are similar to that in Fig. 1. The risk incidences under the leaky vaccine are close to that in Fig. 2.

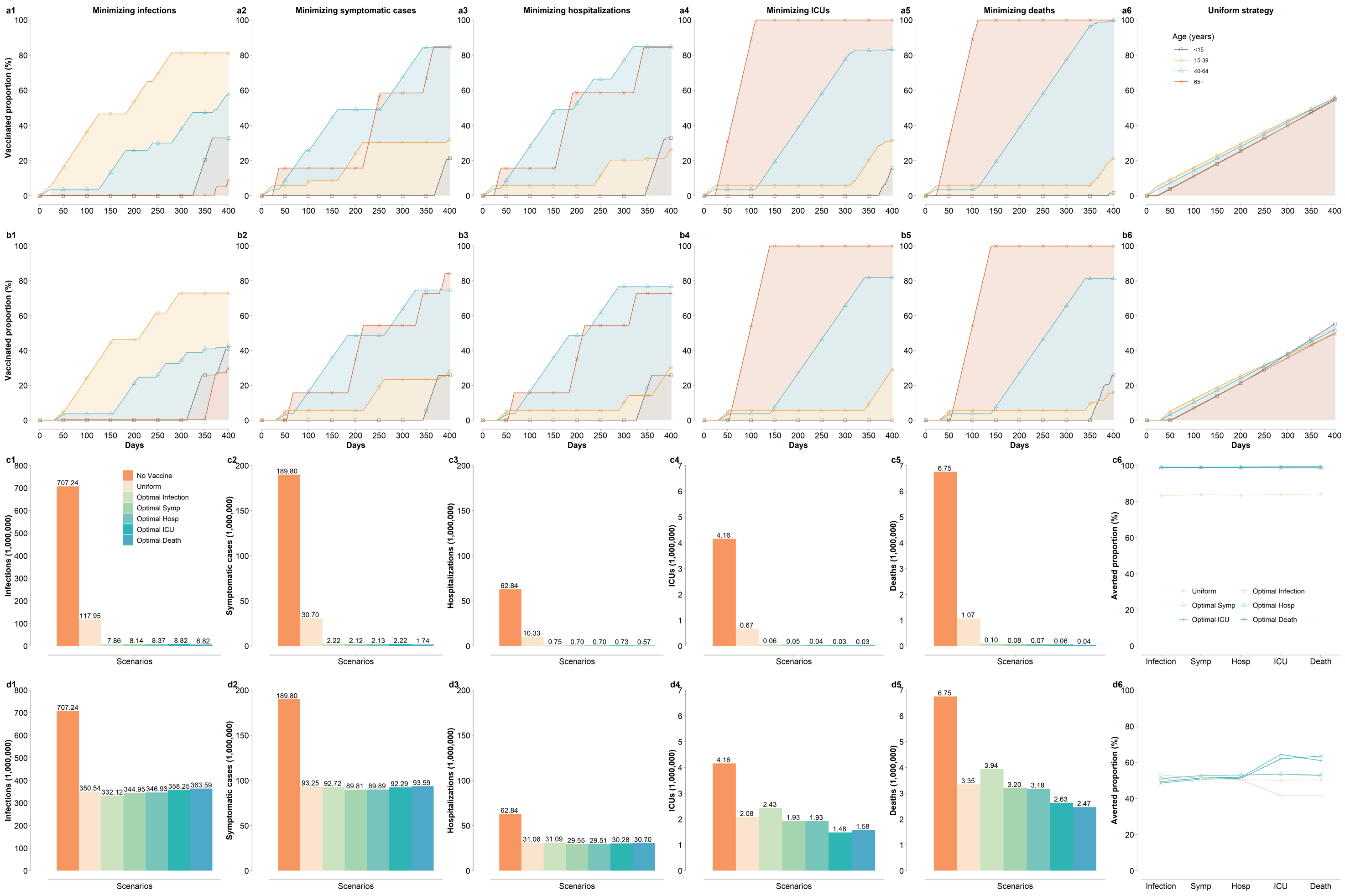

Supplementary Fig. 13: Prioritization strategies and risk incidences under differential timings of vaccination campaign relative to the epidemic (rollout speed 0.14% and R = 1.5). a1 and **b1**, The optimal prioritization strategy for minimizing infections when vaccination start 30 days prior to epidemic onset or 30 days post to epidemic onset respectively. **a2-a6** and **b2-b6**, As panel **a1** and **b1** respectively, but for minimizing symptomatic cases, hospitalizations, ICUs and deaths, and the uniform strategy. **c1-c6** and **d1-d6**, Risk incidences and averted proportions under the two scenarios respectively. The optimal prioritization strategies are similar to that in Fig. 1. The benefits of optimal prioritization strategies are negligible when vaccination start 30 days post to epidemic onset. Keys in the first row apply to all panels in the first two rows, keys in the third row apply to all panels in the last two rows.

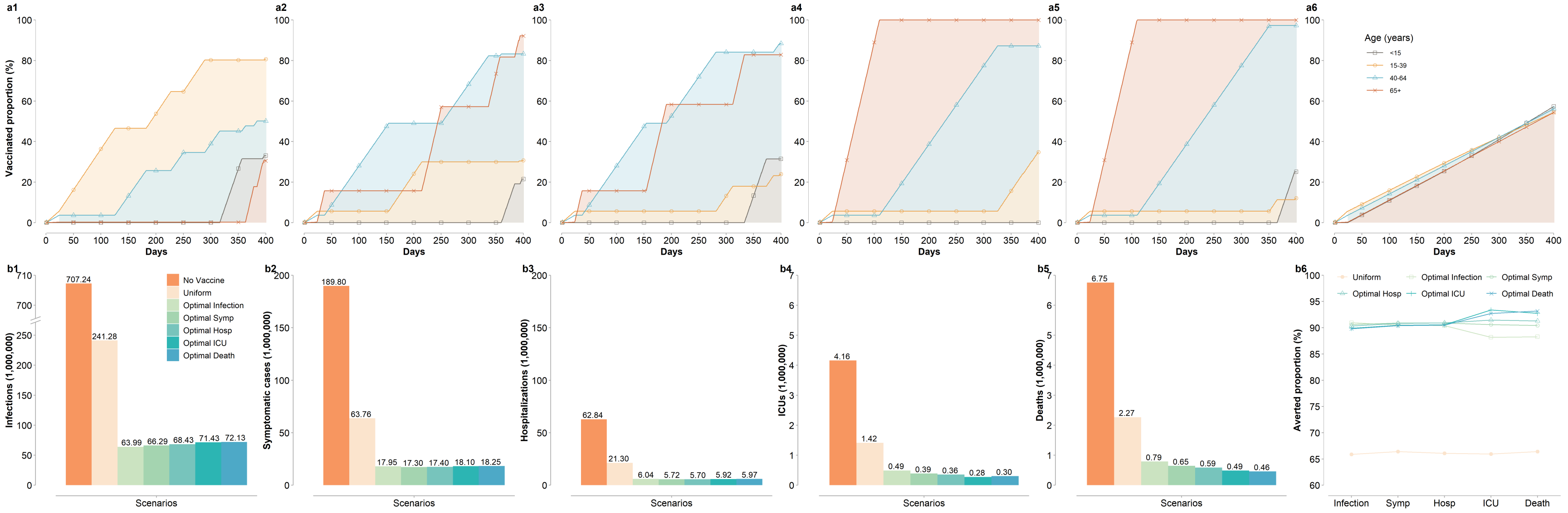

Supplementary Fig. 14: Prioritization strategies and risk incidences with uncertainty in reported cases (rollout speed 0.14% and R = 1.5). a1, The optimal prioritization strategy for minimizing infections after accounting for uncertainty on infections and 11 days of reported lags. **a2-a6**, As **a1**, but for minimizing symptomatic cases, hospitalizations, ICUs and deaths, and the uniform strategy. **b1-b6**, Risk incidences and averted proportions under the scenario. The optimal prioritization strategies are similar to that in Fig. 1. The benefits of optimal prioritization strategies are similar to that in Fig. 2. Keys apply to all panels in the same row.

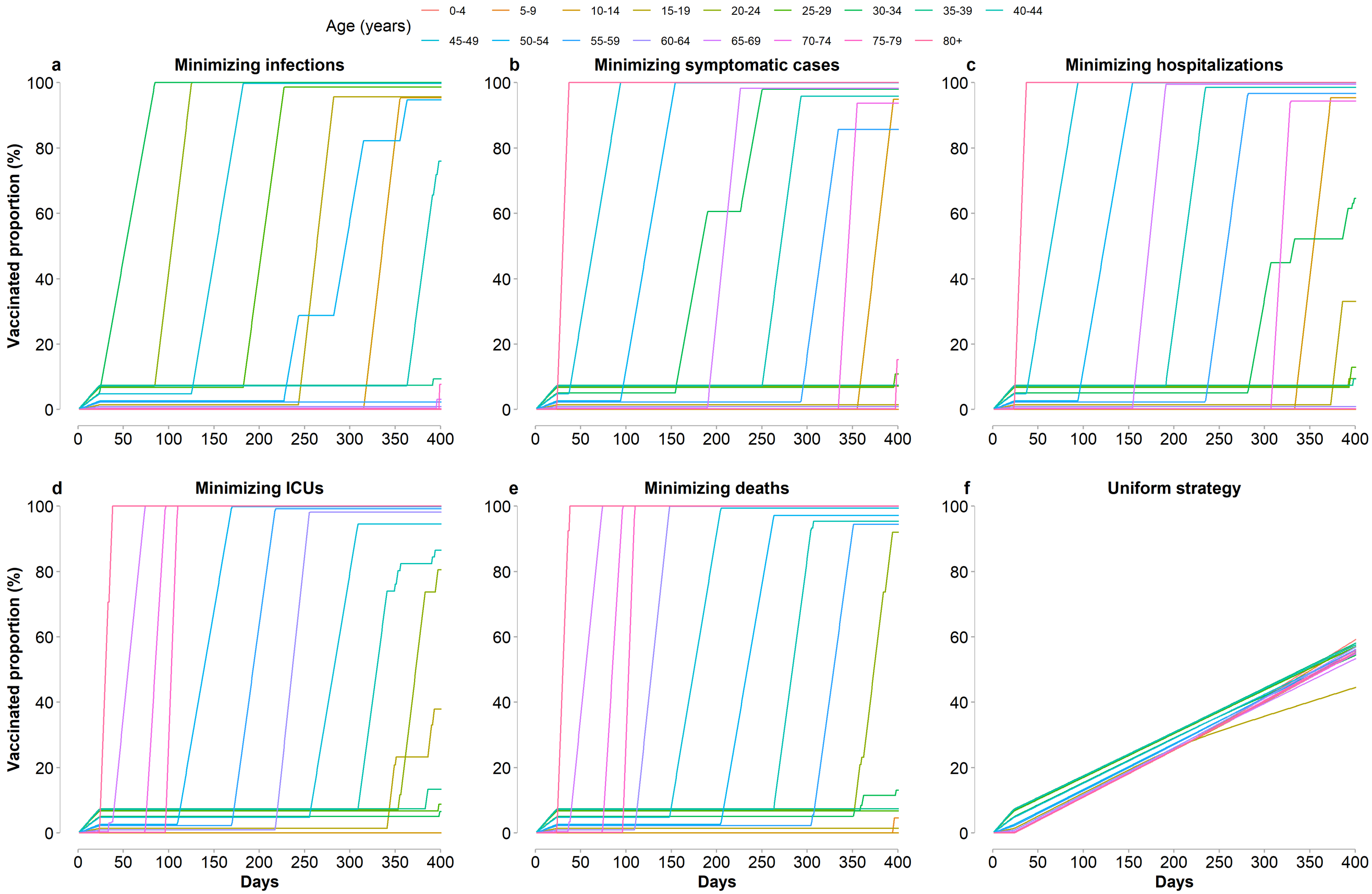

Supplementary Fig. 15: Vaccinated proportions over time in the baseline. a-e, Five optimal prioritization strategies for minimizing infections, symptomatic cases, hospitalizations, ICUs and deaths. **f**, The uniform strategy. Under the uniform strategy, vaccines are allocated proportionally to the size of unvaccinated susceptible populations, which changes over time. Keys and labels apply to all panels.

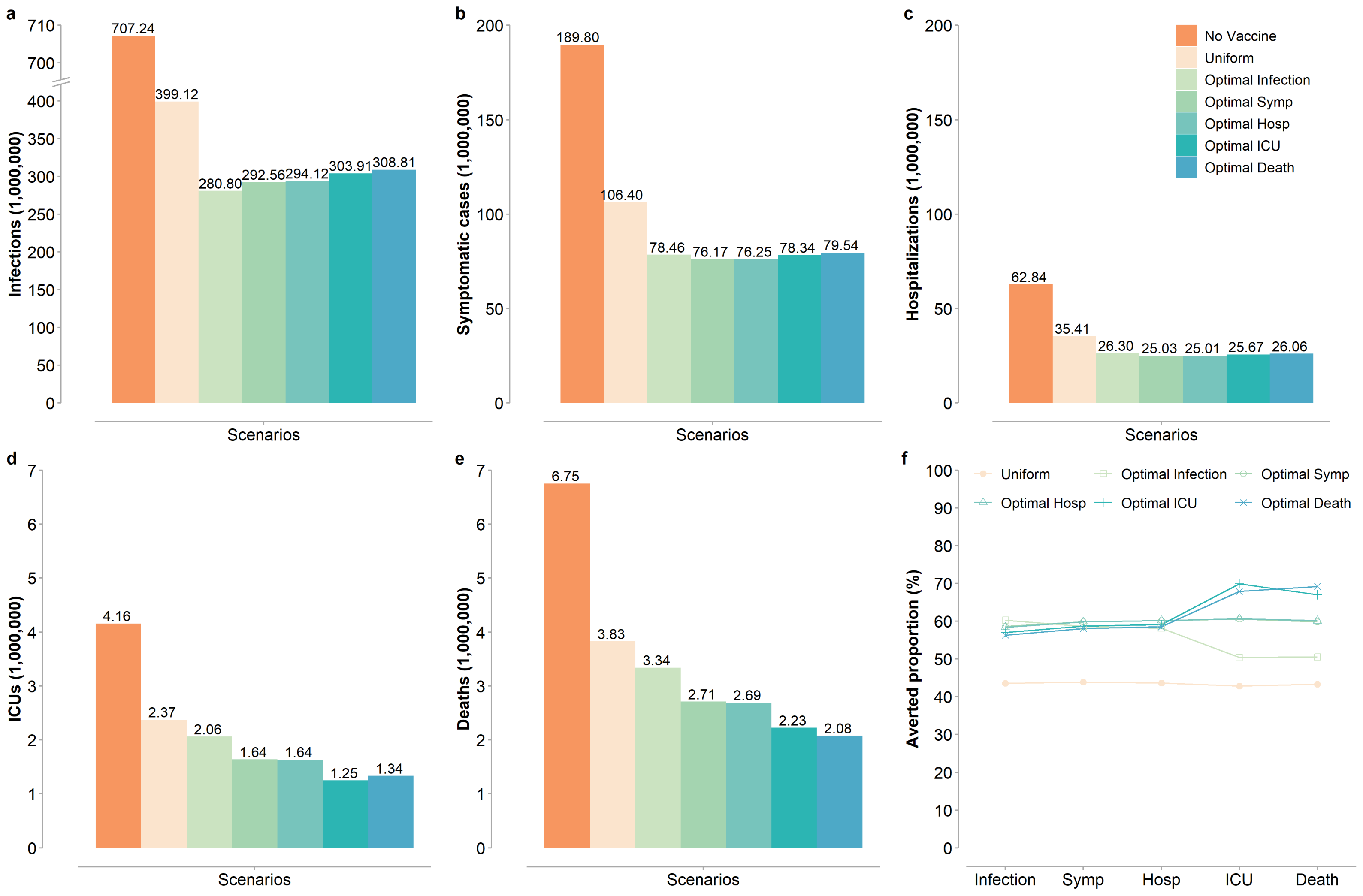

Supplementary Fig. 16: Risk incidences under increased daily rollout speed. Rollout speed increases from 0.10% (1.5 million courses) to 0.17% (2.5 million courses) an average 0.14% (2.0 million courses). **a**, Number of infections under the increased daily rollout speed. **b-e**, As for **a**, but for the number of symptomatic cases, hospitalizations, ICUs and deaths. **f**, Averted risk proportions of the six vaccination policies in comparison to the scenario with no vaccination. All strategies have more infections relative to the baseline. Advantage optimal prioritization strategies over the uniform strategy decreases. Findings apply to all the five risk measures. Keys apply to all panels.

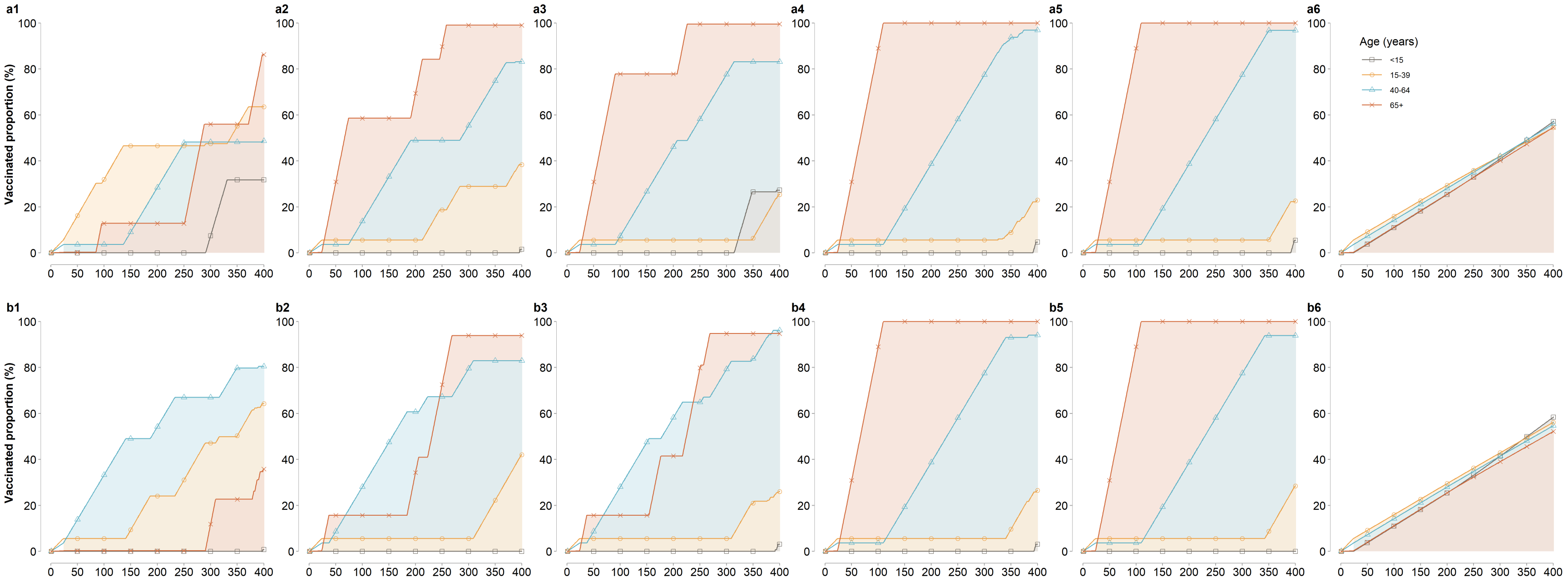

Supplementary Fig. 17: Prioritization strategies under homogenous vaccine efficacy and alternative age-mixing pattern. a1, Optimal prioritization strategy for minimizing infections under homogeneous 80% vaccine efficacy for all age groups. **b1**, As **a1**, but under pandemic age-mixing patters (Supplementary Fig. 21b). **a2**-**a6** and **b2**-**b6**, As panel **a1**and **b1** respectively, but for minimizing symptomatic cases, hospitalizations, ICUs, deaths and the random mass vaccination. **a1**, adults over 65, the non-priority group in baseline (Fig. 1), are identified as the second highest priority with small coverages.

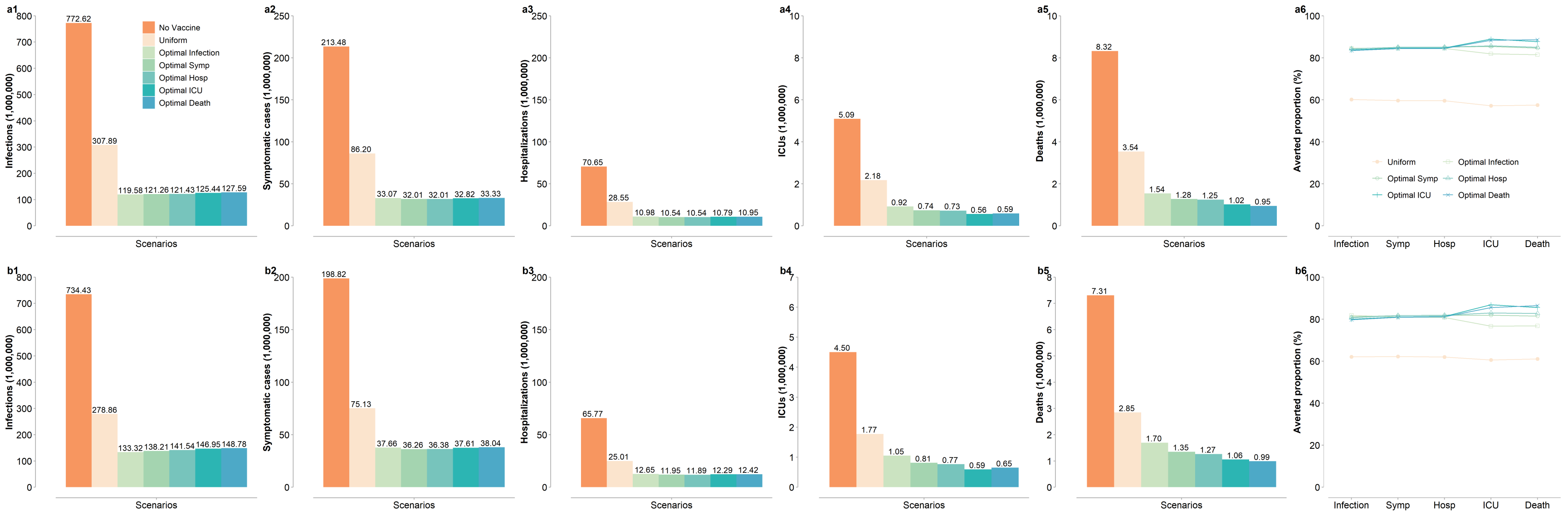

Supplementary Fig. 18: Risk incidences and reductions under alternative contact matrix. a1, Number of infections under pandemic contact patterns (Supplementary Fig. 21b). **b1**, As **a1**, but under the adjusted infectiousness (Supplementary Fig. 21c), where symptomatic infections are twice as infectiousness as asymptomatic case. **a2-a6** and **b2-b6**, As panel **a1** and **b1** respectively, but for the number of symptomatic cases, hospitalizations, ICUs, and deaths, as well as averted proportions. Keys in **a1** apply to all bar plots. Keys in **a6** apply to all line plots.

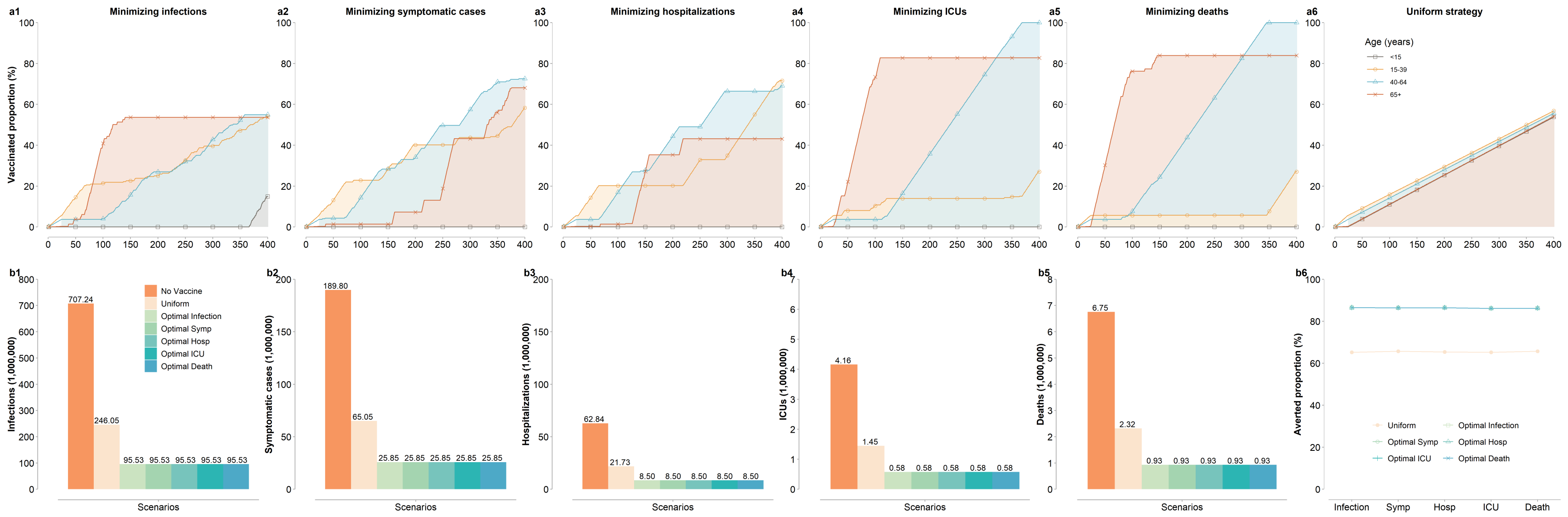

Supplementary Fig. 19: Prioritization strategies and risk incidences when vaccination campaign includes all previously infected people. a1 and **b1**, the optimal prioritization strategy for minimizing infections. **a2-a6** and **b2-b6**, As panel **a1** and **b1,** respectively, but for minimizing symptomatic cases, hospitalizations, ICUs and deaths, and the uniform strategy. **b1-b6**, risk incidences and averted proportions under the scenario. Keys in the first row apply to all panels in the first rows, keys in panel **b** apply to bar plots. Note that the optimal prioritized allocation strategy differs from that estimated in the baseline analysis.

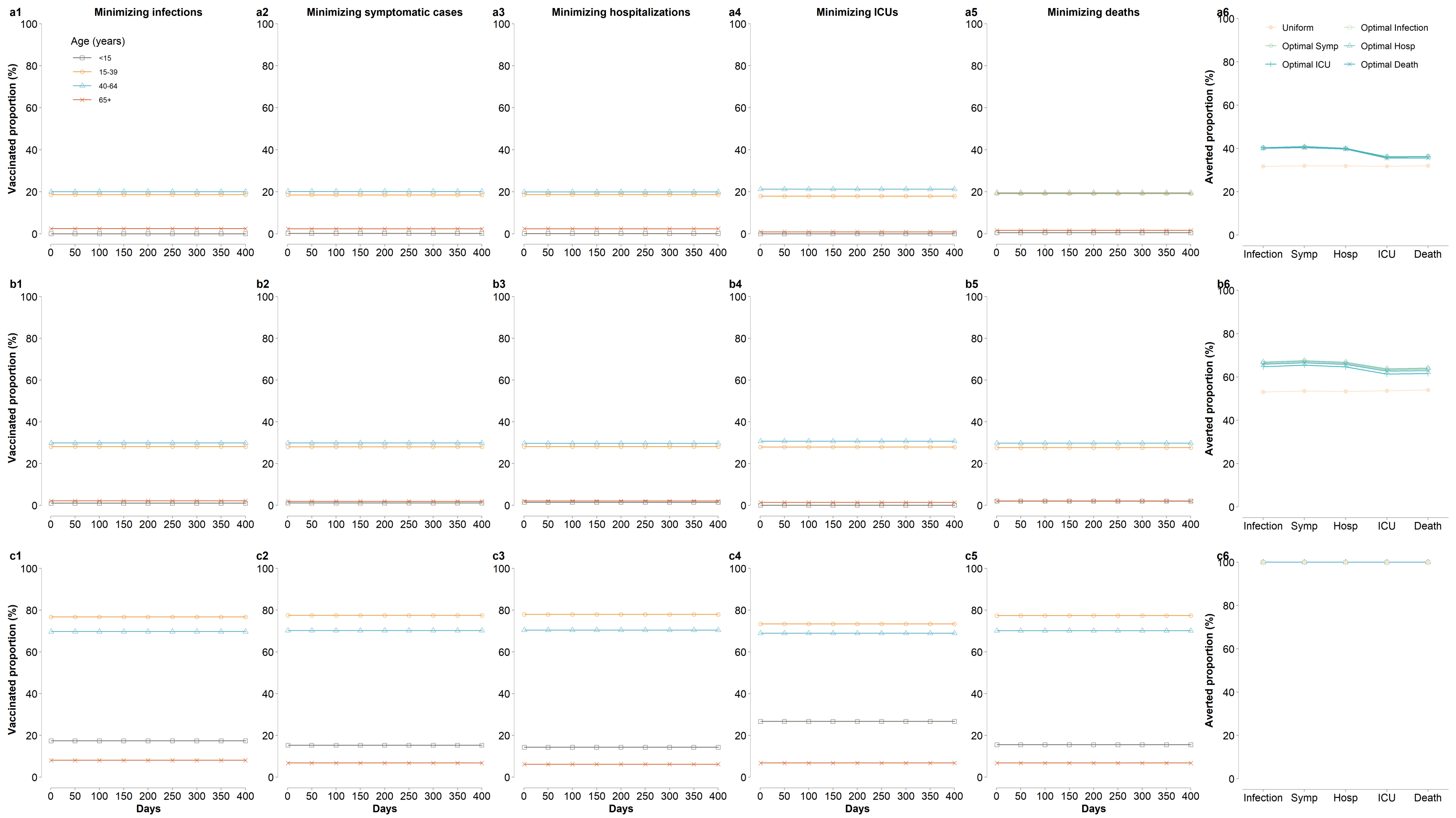

Supplementary Fig. 20: Allocation strategies and risk reductions for scenarios where vaccination campaigns complete prior to the epidemic. a1, Allocation strategies when minimizing infections with a total of 2 × 100 million vaccines. **b1**, Allocation strategies when minimizing infections with a total of 2 × 150 million vaccines. **c1**, Allocation strategies when minimizing infections with a total of 2 × 400 million vaccines. **a2**-**a6**, **b2**-**b6** and **c2**-**c6**, As panel **a1**, **b1** and **c1** respectively, but for the number of symptomatic cases, hospitalizations, ICUs, and deaths, as well as a summary of averted proportions. Keys in **a1** apply to all line plots without dot marks. Keys in **a6** apply to all line plots with dot marks. Because vaccination campaign completes prior to the epidemic, the vaccination coverage is fixed as the epidemic unfolds.

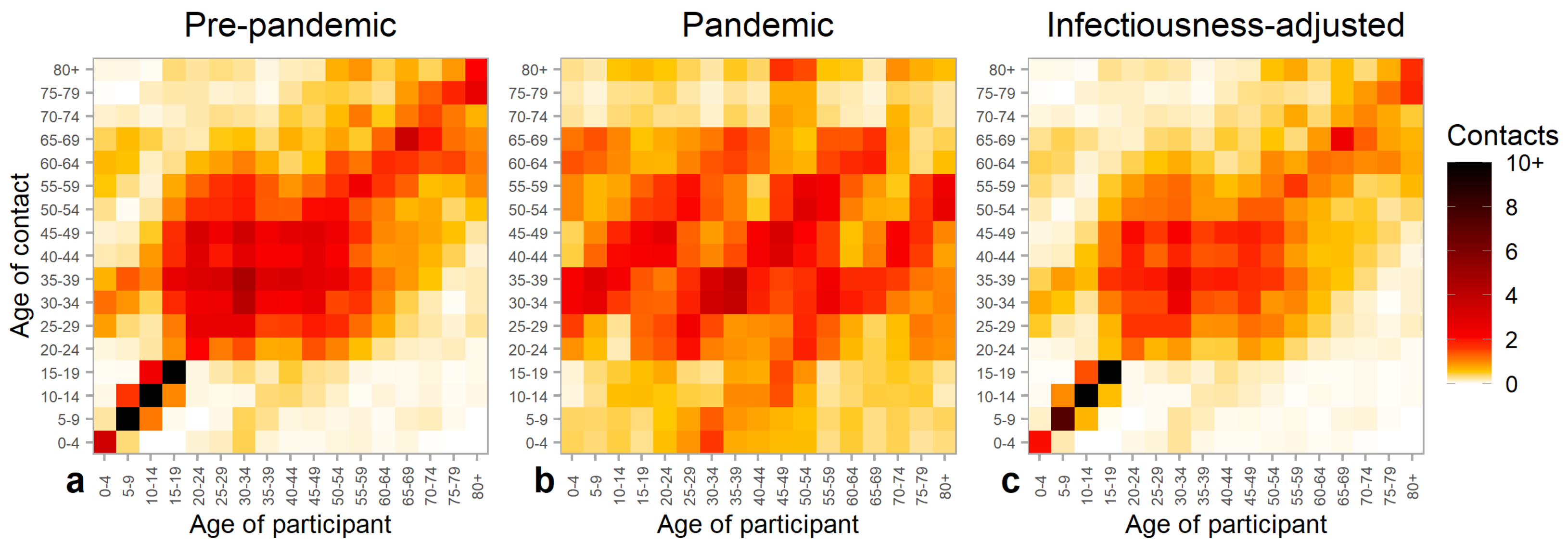

Supplementary Fig. 21: Contact matrices by age in China. a, Pre-pandemic contact matrix, which is used in the main analysis. Mixing patterns were derived from literature [2] and refers to Shanghai, China in 2017/2018. Each cell of the matrix represents the mean number of contacts that an individual in a given age group has with other individuals, stratified by age groups. The color intensity represents the number of contacts. **b**, Pandemic contact matrix. Mixing patterns were derived from literature [3] and refers to Shanghai, China in March 2020, when interventions were relaxed after the lockdown. **c**, Infectiousness-adjusted contact matrix. Here the pre-pandemic contact matrix is adjusted to account for a hypothetical reduction of 50% of the infectiousness of asymptomatic individual. Specifically, since the probability of developing symptoms depends on age [4,5], this adjustment does not correspond to applying a single scale factor.

Supplementary Table 1 Summary of parameters

Parameter	Description	Baseline value	Reference
J	Number of age groups	17	
N_i	Population size for age group *i*	Supplementary Table 2	[6]
$N_{i,k}$	Population size for age group *i* within tier *k*	Supplementary Table 2	[7,8]
β	Transmission rate	Derived from *R* through Equation (3)	[9]
$1/\gamma$	Generation time	5.5 days	[10]
$C_{i,j}$	Contact matrix	Supplementary Fig. 21a	[2]
s_i	Relative susceptibility to SARS-CoV-2 infection for age group *i*	Supplementary Table 2	[10]
e_i	Vaccine efficacy (protection against the infection) for age group i, $i = 4,.., 12$	80%	[11]
	Vaccine efficacy (protection against the infection) for age group i, $i \leq 3$, or $i \geq 13$	0.75×80%	[9,12]
$1/w$	Delay from the administration of the first vaccine dose and protection	21+14 days	[13]
r_i^{symp}	Risk of developing symptoms given the infection for age group *i*	Supplementary Table 2	[4]
r_i^{hosp}	Risk of requiring hospitalization given the infection for age group *i*	Supplementary Table 2	[4,5]
r_i^{icu}	Risk of requiring ICU given the infection for age group *i*	Supplementary Table 2	[4,5,14]
r_i^{death}	Infection fatality risk for age group *i*	Supplementary Table 2	[4,5]
c	Daily vaccination capacity of first doses	2.0 million	[15]
$a_{i,1}$	Vaccine acceptance for age group *i* within tier *1*	Supplementary Table 2	Author H.Y. unpublished results
$a_{i,2}$	Vaccine acceptance for the general population for age group *i* within tier *2*	Supplementary Table 2	Author H.Y. unpublished results

Supplementary Table 2 Population demographics and risk distributions

Age group (years)		Population size in tier 1	Population size in tier 2	Total population size	Relative susceptibility to infection	Vaccine acceptance (general population)	Risk of developing symptoms	Risk of requiring hospitalization	Risk of requiring ICU admission	Risk of death
**Reference**		7,8	7,8	6	10	Unpublished results	4	4,5	4,5,14	4,5
	i	$N_{i,1}$	$N_{i,2}$	N_i	s_i	$a_{i,2}$	r_i^{symp}	r_i^{hosp}	r_i^{icu}	r_i^{death}
**0-4**	1	0	83,932,437	83,932,437	0.580	0.810	0.181	0.072	0.000	0.001
**5-9**	2	0	86,735,183	86,735,183	0.580	0.810	0.181	0.072	0.000	0.001
**10-14**	3	0	84,262,751	84,262,751	0.580	0.850	0.181	0.072	0.000	0.001
**15-19**	4	1,181,745	81,160,114	82,341,859	1.000	0.850	0.181	0.072	0.002	0.001
**20-24**	5	6,217,027	80,941,140	87,158,167	1.000	0.810	0.224	0.065	0.001	0.001
**25-29**	6	6,635,693	91,353,310	97,989,003	1.000	0.810	0.224	0.065	0.001	0.001
**30-34**	7	6,496,402	122,242,568	128,738,970	1.000	0.780	0.224	0.065	0.001	0.001
**35-39**	8	7,379,797	92,711,658	100,091,455	1.000	0.780	0.224	0.065	0.001	0.001
**40-44**	9	6,906,755	89,367,391	96,274,146	1.000	0.840	0.305	0.102	0.002	0.007
**45-49**	10	5,733,610	114,104,007	119,837,617	1.000	0.840	0.305	0.102	0.002	0.007
**50-54**	11	3,264,296	120,181,086	123,445,382	1.000	0.840	0.305	0.102	0.007	0.007
**55-59**	12	2,214,108	96,526,383	98,740,491	1.000	0.840	0.305	0.102	0.007	0.007
**60-64**	13	711,933	76,802,206	77,514,139	1.000	0.890	0.355	0.120	0.009	0.037
**65-69**	14	295,280	73,854,486	74,149,766	1.650	0.890	0.355	0.120	0.025	0.037
**70-74**	15	33,563	44,842,807	44,949,689	1.650	0.780	0.355	0.120	0.025	0.037
**75-79**	16	48,461	26,496,155	26,544,616	1.650	0.780	0.355	0.120	0.025	0.037
**80+**	17	0	26,618,103	26,618,103	1.650	0.780	0.646	0.218	0.046	0.068

References

1. Lazarus, J. V *et al.* A global survey of potential acceptance of a COVID-19 vaccine. *Nature medicine* **27**, (2020).
2. Zhang, J. *et al.* Patterns of human social contact and contact with animals in Shanghai, China. *Scientific Reports* **9**, (2019).
3. Zhang, J. *et al.* The impact of relaxing interventions on human contact patterns and SARS-CoV-2 transmission in China. *Science Advances* **7**, eabe2584 (2021).
4. Poletti, P. *et al.* Association of Age With Likelihood of Developing Symptoms and Critical Disease Among Close Contacts Exposed to Patients With Confirmed SARS-CoV-2 Infection in Italy. *JAMA Network Open* **4**, e211085 (2021).
5. Yang, J. *et al.* Disease burden and clinical severity of the first pandemic wave of COVID-19 in Wuhan, China. *Nature communications* **11**, 1–10 (2020).
6. World Population Prospects 2019- Population Division - United Nations. https://population.un.org/wpp/.
7. Yang, J. *et al.* Who should be Prioritized for COVID-19 Vaccination in China? A Descriptive Study. *BMC Medicine* **19**, (2020).
8. Wang, W. *et al.* Global, regional, and national estimates of target population sizes for COVID-19 vaccination. *BMJ* **15**, (2020).
9. Yang, P. *et al.* Influenza vaccine effectiveness against medically-attended influenza illness during the 2012-2013 season in Beijing, China. *Vaccine* **32**, 5285–5289 (2014).
10. Hu, S. *et al.* Infectivity, susceptibility, and risk factors associated with SARS-CoV-2 transmission under intensive contact tracing in Hunan, China. *Nat. Commun.* **12**, (2021).
11. Kaabi, N. Al *et al.* Effect of 2 Inactivated SARS-CoV-2 Vaccines on Symptomatic COVID-19 Infection in Adults: A Randomized Clinical Trial. *JAMA* **326**, 35–45 (2021).
12. Xia, S. *et al.* Safety and immunogenicity of an inactivated SARS-CoV-2 vaccine, BBIBP-CorV: a randomised, double-blind, placebo-controlled, phase 1/2 trial. *The Lancet Infectious Diseases* **21**, (2020).
13. China National Biotec Group Company Limited (2020-10-30). *A study to evaluate the efficacy, safety and immunogenicity of inactivated SARS-CoV-2 vaccines (vero cell) in healthy population aged 18 years old and above (COVID-19).* https://clinicaltrials.gov/ct2/show/NCT04510207.
14. Guan, W. *et al.* Clinical characteristics of coronavirus disease 2019 in China. *New England journal of medicine* **382**, 1708–1720 (2020).
15. COVID-19 vaccines vaccination status. http://www.gov.cn/xinwen/2021-05/09/content_5605500.htm.
